# Tumor-specific expression of HMG-CoA reductase in a population-based cohort of breast cancer patients

**DOI:** 10.1186/s12907-015-0008-2

**Published:** 2015-05-20

**Authors:** Emma Gustbée, Helga Tryggvadottir, Andrea Markkula, Maria Simonsson, Björn Nodin, Karin Jirström, Carsten Rose, Christian Ingvar, Signe Borgquist, Helena Jernström

**Affiliations:** Division of Oncology and Pathology, Department of Clinical Sciences, Lund, Lund University, Barngatan 2B, SE 22185 Lund, Sweden; Department of Oncology, Skåne University Hospital, Lund, Sweden; CREATE Health and Department of Immunotechnology, Lund University, Medicon Village, Building 406, Lund, Sweden; Department of Clinical Sciences, Division of Surgery, Lund, Lund University, Lund, Sweden and Skåne University Hospital, Lund, Sweden

**Keywords:** Breast cancer, HMG-CoA reductase, Tumor characteristics, Treatment, Early breast cancer events, Prognosis

## Abstract

**Background:**

The mevalonate pathway synthetizes cholesterol, steroid hormones, and non-steriod isoprenoids necessary for cell survival. 3-Hydroxy-3-methylglutaryl-coenzyme A reductase (HMGCR) is the rate-limiting enzyme of the mevalonate pathway and the target for statin treatment. HMGCR expression in breast tumors has recently been proposed to hold prognostic and treatment-predictive information. This study aimed to investigate whether HMGCR expression in breast cancer patients was associated with patient and tumor characteristics and disease-free survival (DFS).

**Methods:**

A population-based cohort of primary breast cancer patients in Lund, Sweden was assembled between October 2002 and June 2012 enrolling 1,116 patients. Tumor tissue microarrays were constructed and stained with a polyclonal HMGCR antibody (Cat. No HPA008338, Atlas Antibodies AB, Stockholm, Sweden, diluted 1:100) to assess the HMGCR expression in tumor tissue from 885 patients. HMGCR expression was analyzed in relation to patient- and tumor characteristics and disease-free survival (DFS) with last follow-up June 30^th^ 2014.

**Results:**

Moderate/strong HMGCR expression was associated with less axillary lymph node involvement, lower histological grade, estrogen and progesterone receptor positivity, HER2 negativity, and older patient age at diagnosis compared to weak or no HMGCR expression. Patients were followed for up to 11 years. The median follow-up time was 5.0 years for the 739 patients who were alive and still at risk at the last follow-up. HMGCR expression was not associated with DFS.

**Conclusion:**

In this study, HMGCR expression was associated with less aggressive tumor characteristics. However, no association between HMGCR expression and DFS was observed. Longer follow-up may be needed to evaluate HMGCR as prognostic or predictive marker in breast cancer.

## Introduction

New prognostic and treatment-predictive markers are needed to improve treatment decisions and consequently prognosis and treatment response in breast cancer patients. Recent data suggest that the enzyme 3-hydroxy-3-methylglutaryl-coenzyme A reductase (HMGCR), which is inhibited by statins that are commonly used as a cholesterol-lowering treatment, may be associated with breast tumor characteristics, prognosis and treatment response [1–3]. HMGCR is the rate-limiting enzyme in the mevalonate pathway [4]. The mevalonate pathway produces cholesterol, steroid hormones, and non-steroid isoprenoids, which are necessary for cell survival [5]. Previous studies demonstrated that HMGCR inhibitors (e.g., statins) exert anti-carcinogenic effects by inducing apoptosis and inhibiting inflammation [6], proliferation and migration [7–9]. HMGCR inhibitors can also inhibit angiogenesis [10]. Whether statins also reduce the risk of cancer remains debated [11–17]. HMGCR is differentially expressed among breast cancers, as well as between normal epithelial cells and tumor cells, with higher expression in the tumor cells. This difference is presumably caused by resistance against the feedback system of the mevalonate pathway [18]. Several studies examined the relationship between tumor-specific HMGCR expression and other tumor characteristics [1, 2]. In previous studies, stronger expression of HMGCR was associated with a less aggressive tumor profile, such as a low histological grade, a small tumor size, estrogen receptor (ER) positivity, and low proliferation [1, 2]. One study reported that patients with HMGCR-positive tumors exhibited longer recurrence-free survival, which was more pronounced in patients with ER-positive tumors [2]. Another study observed longer recurrence-free survival in patients treated with tamoxifen who had HMGCR-positive and ER-positive tumors compared to patients who had ER-positive and HMGCR-negative tumors, indicating that HMGCR may predict tamoxifen response [3].

### Hypothesis and aim

We hypothesized that stronger HMGCR expression was associated with markers of good prognosis and prolonged disease-free survival (DFS), as well as a better response to tamoxifen, in this population-based unselected cohort of primary breast cancer patients. The aims of the study were to investigate whether HMGCR expression in breast cancer was associated with patient and tumor characteristics, prognosis and treatment response.

## Materials and methods

### Patients

Women diagnosed with a primary breast cancer at Skåne University Hospital in Lund, Sweden between October 2002 and June 2012 were invited to take part in an ongoing prospective cohort study: the Breast Cancer (BC) Blood study. During the inclusion period, 1,116 patients were included in the study and followed-up until June 30^th^, 2014. Patients with a previous breast cancer diagnosis or another cancer diagnosis during the previous ten years were not included. The aim was to study factors that could affect prognosis or treatment response and to identify new markers that may help better tailor adjuvant therapy to individual breast cancer patients. The study was approved by Lund University Ethics Committee (Dnr LU75-02, LU37-08, LU658-09, LU58-12, and LU379-12). All patients signed a written informed consent. The study adhered to the REMARK criteria [19].

All patients completed questionnaires preoperatively. Post-operative questionnaires were completed after 3–6 months, 7–9 months, and 1, 2, 3, 5, 7, 9 and 11 years. The questionnaires included questions concerning medication intake during the last week, lifestyle, and reproductive factors. Medications were coded according to the Anatomic Therapeutic Chemical (ATC) classification system codes [20]. Patients who reported smoking during the last week or smoking at parties were considered current smokers. Coffee consumption was categorized as 0–1 or 2+ cups/day, as previously described [21]. A research nurse obtained body measurements, including, height, weight, waist and hip circumferences, and breast volumes, at the pre-operative visit. Breast volume was measured as previously described [22, 23].

Tumor characteristics were acquired from the patients’ pathology reports. The estrogen receptor (ER) and progesterone receptor (PgR) expression were analyzed in the Department of Pathology at Skåne University Hospital in Lund, Sweden. Until December 2009, immunohistochemistry was performed using the Dako LSAB kit system (Dako, Glostrup, Denmark) and the M7047 (ER) and M3569 (PgR) antibodies (DAKO) [24, 25]. From 2010 onwards, the ER (SP1) and PgR (1E2) antibodies from Ventana Medical Systems (Ventana, AZ, USA) were used in combination with a Ventana Benchmark Ultra instrument (Ventana Medical Systems) [26]. Tumors with more than 10 % positive nuclear staining were considered ER-positive or PgR-positive according to current Swedish clinical guidelines. Histological type was classified into ductal, lobular and ‘other’ types. Ten tumors had a mixed ductal and lobular histology and were classified as ‘other’. Since the tumors were not routinely analyzed for HER2 amplification until November 2005, patients included in the study prior to that time were excluded from analyses that included HER2 status.

**Fig. 1:**
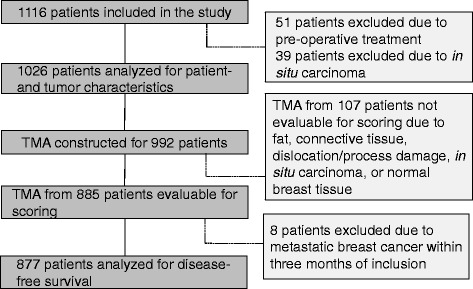
Flow chart of the patient selection process

Information on type of surgery, treatment, and breast cancer related events was obtained from patient charts and the regional tumor registry. The date of death was collected from the Swedish Population Registry. Patients who had received preoperative treatment (n = 51) and patients with cancer *in situ* (n = 39) were excluded from the analyses, leaving 1,026 preoperatively untreated patients with invasive breast cancer as the study population (Fig. 1).

### Tissue microarray construction

Tumor tissue was available from 992 of the 1,026 patients. Tissue microarrays (TMAs) for the tumors were constructed by sampling 1 mm duplicate cores from representative, non-necrotic tumor regions from the donating formalin-fixed paraffin-embedded tumor tissue block from surgical resection, using a semi-automated tissue array device (Beecher Instruments, Sun Prairie, WI, USA).

### Immunohistochemistry

**Fig. 2:**
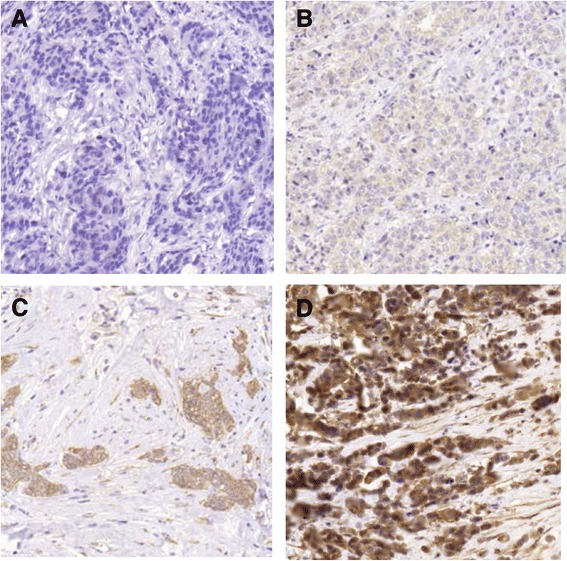
Examples of HMGCR expression, representing no staining (**a**), and weak (**b**), moderate (**c**), and strong (**d**) expression. The original magnification before scale-down was 20x for each example

An automatic PT-link system (DAKO, Glostrup, Denmark) was used to deparaffinize and pretreat 4 μm TMA-sections for HMGCR staining. HMGCR staining was performed using an Autostainer Plus, according to the manufacturer’s instructions (DAKO). The staining procedure employed an HMGCR antibody (Cat. No HPA008338, Atlas Antibodies AB, Stockholm, Sweden) (diluted 1:100) and an EnVision FLEX high-pH kit. HMGCR expression could be evaluated in tumors from 885/992 patients. In 57 cases, the TMA-cores contained non-representative tissue, in 27 cases, the cores were damaged or lost during processing, and in 23 cases, the cores could not be evaluated due to a combination of the reasons mentioned above. HMGCR expression was evaluated based on the staining intensity in the cytoplasm (i.e., negative = 0, weak = 1, moderate = 2, and strong = 3), as shown in Fig. 2, and based on the fraction of HMGCR-positive cells (0 % = 0, 1-10 % = 1, 11-50 % = 2, 51-100 % = 3). Two investigators, who were blinded to the patient data and clinical outcome, evaluated all samples simultaneously (EG, HT). When the two investigators could not reach a consensus, a senior investigator (SB) was consulted and a consensus was reached. The HMGCR expression differed between the duplicate cores for 109 patients. In all cases but one, the intensity only differed by one step. Discordant cores were reevaluated jointly to obtain a pooled score based on the intensity represented in the majority of cancer cells. When one core was classified as negative and the other core was classified as positive, the pooled score was classified as positive. Only 22 tumors showed strong intensity of HMGCR expression, and this group was combined with tumors expressing HMGCR with a moderate intensity (n = 195). A total of 28 of the 1,026 patients had bilateral tumors; tissue from both tumors was available for 15 patients. Scoring of both bilateral tumors was possible for 10 of these patients. For the three cases where the intensity differed, the highest intensity was used. In most cases (94.9 %) for which the staining was positive in any cell, HMGCR was expressed in the majority of the cells (51-100 %). Therefore, the fraction of HMGCR-positive cells was excluded from further analyses.

### Statistics

The statistical analyses were performed using SPSS Statistics 19 (IBM, Chicago, IL, USA). Patient and tumor characteristics were analyzed in relation to HMGCR expression. The Chi-square and Linear-by-Linear tests were used for categorical variables. The Kruskal-Wallis and Jonckheere-Terpstra tests were used for continuous variables because some of these variables were not normally distributed. Tumor characteristics in relation to HMGCR tumor expression were also analyzed with linear regression and adjusted for age as a continuous variable. The DFS was calculated from the date of inclusion until the first breast cancer related event (i.e., local or regional recurrence, contralateral breast cancer, or distant metastasis); in cases with no breast cancer related events, DFS was calculated using the last study follow-up or death before July 1^st^, 2014. Non-breast cancer-related death was censored at the time of death. Patients with distant metastases detected earlier than three months after inclusion were excluded from the survival analyses (n = 8). Univariable survival analyses were calculated using Log-Rank tests. Cox proportional hazard regression was used for multivariable testing, with adjustments for invasive tumor size (>20 mm or muscular or skin involvement), axillary lymph node involvement, histological grade (III), ER and PgR status (positive/negative), age <50 years (yes/no), current preoperative smoking (yes/no), and body mass index (BMI) <25 kg/m^2^ (yes/no) [27]. All statistical tests were two-tailed. *P*-values < 0.05 were regarded as statistically significant. Nominal *P*-values were presented without adjustment for multiple testing.

## Results

### Patient characteristics and HMGCR expression

The patient characteristics are presented in relation to the HMGCR expression (Table 1). Of the 885 cases evaluable for scoring, the intensity of HMGCR expression was negative in 113 cases (12.8 %), weak in 555 cases (62.7 %), and moderate/strong in 217 (24.5 %) cases. Patients of all ages were included (range 24–99 years). The median age at inclusion in the study was 61.1 years (interquartile range 52.1 to 68.1). Patients younger than 50 years at inclusion were significantly taller, had a lower BMI, and had smaller waist and hip circumferences, waist-to-hip ratios, and breast volumes than older patients (*P* ≤ 0.001 for all comparisons); however, these patients had a weight similar to that of older patients. The final surgery performed included partial mastectomy in 531 cases (60 %) and modified radical mastectomy in 354 cases (40 %). Postoperative radiotherapy was given to 559 patients (63.2 %) and 226 patients (25.5 %) received adjuvant chemotherapy. As adjuvant endocrine therapy, tamoxifen treatment was prescribed to 466 patients (52.7 %) and 302 patients (34.1 %) were treated with aromatase inhibitors. As of November 2005, 55 patients (8.6 %) had received adjuvant treatment with trastuzumab (n = 640). Patients often received more than one type of treatment.

**Table 1:** Association of HMGCR expression with patient characteristics

	All patients median (IQR) or %	Missing	Patients with evaluable TMA	HMGCR expression-Intensity median (IQR) or %			
				No staining = 0	Weak = 1	Moderate/Strong = 2/3	*P*-value
n=	1026		885	113	555	217	
**Age at inclusion**, **yrs**	61.1 (52.1-68.1)	0	60.9 (52.2-67.9)	61.2 (53.7-69.2)	60.1 (51.6-67.4)	64.3 (53.9-69.4)	0.004^c^
**Body mass index** **(BMI), (kg/m-2)**	25.1 (22.5-28.3)	28	25.2 (22.5-28.4)	25.7 (23.2-28.7)	25.0 (22.4-28.3)	25.3 (22.5-29.0)	0.39^c^
**Height (cm)**	165 (162–170)	26	165 (162–170)	164 (160–169)	166 (162–170)	165 (161–169)	0.03^c^
**Weight (kg)**	69 (62–78)	26	69 (62–78)	70 (62–79)	69 (62–78)	69 (61–80)	0.91^c^
**Waist**-**Hip**-**Ratio**	0.86 (0.81-0.9)	38	0.86 (0.81-0.90)	0.86 (0.81-0.92)	0.85 (0.80-0.90)	0.86 (0.81-0.90)	0.32^c^
**Waist (cm)**	87 (79–97)	38	87 (80–97)	89 (82–97)	87 (79–97)	89 (80–98)	0.24^c^
**Hip (cm)**	102 (97–109)	38	102 (97–109)	104 (99–109)	102 (97–109)	102 (96–111)	0.57^c^
**Breast volume (ml)**	1000 (650–1500)	161	1000 (650–1550)	1000 (700–1550)	950 (640–1500)	1000 (656–1600)	0.28^c^
**Age at menarche, yrs**	13 (12–14)	7	13 (12–14)	13 (13–14)	13 (12–14)	13 (12–14)	0.68^c^
**Nulliparous**	124 (12.1 %)	1	109 (12.3 %)	17 (15.0 %)	67 (12.1 %)	25 (11.5 %)	0.41^d^
**Parity**	2 (1–3)	1	2 (1–3)	2 (1–3)	2 (1–3)	2 (1–2)	0.46^c^
Age at first child, years*	25 (22–28)	6	24 (22–28)	24 (21–27)	25 (21–28)	25 (22–29)	0.09^b^
**Ever use of HRT**	449 (43.9 %)	3	386 (43.8 %)	55 (48.7 %)	231 (41.8 %)	100 (43.8 %)	0.28^a^
**Ever oral contraceptives**	726 (70.8 %)	1	625 (70.7 %)	82 (72.6 %)	394 (71.1 %)	149 (68.7 %)	0.42^d^
**Current smoker**	210 (20.5 %)	2	177 (20.0 %)	26 (23.0 %)	111 (20.1 %)	40 (18.4 %)	0.34^d^
**Coffee**, **2+ cups per day**	832 (81.4 %)	4	708 (80.4 %)	89 (78.8 %)	448 (81.3 %)	171 (78.8 %)	0.66^a^
**Alcohol abstainer**	107 (10.5 %)	7	96 (10.9 %)	15 (13.3 %)	56 (10.1 %)	25 (11.6 %)	0.58^a^

*Among Parous women, IQR = Inter quartile range ^a^Chi-Square, ^b^Jonckheere-Terpstra, ^c^Kruskal-Wallis, ^d^Linear-by-linear

Patients with tumors that expressed moderate/strong HMGCR were significantly older than patients in the other HMGCR intensity groups at inclusion. Patients with tumors that expressed weak HMGCR were significantly taller than patients in the other HMGCR intensity groups. No other significant associations were observed between patient characteristics and HMGCR expression. The results remained essentially the same when excluding patients who reported statin usage preoperatively (n = 99).

### HMGCR expression and established tumor characteristics

Table 2 displays the tumor characteristics in relation to HMGCR expression. Tumor size was not associated with HMGCR expression. Tumors that expressed moderate/strong HMGCR were of significantly lower histological grade, were more frequently ER-positive, PgR-positive, and HER2-negative, and were less likely to show axillary lymph node involvement compared to patients whose tumors had weak or no HMGCR expression. Histological type was not associated with HMGCR expression. HER2 amplification was more common among patients with HMGCR-negative tumors. These associations remained significant after adjustment for age. The results remained essentially the same after exclusion of patients with preoperative statin usage. Ki67 was only available for 365 patients (41.2 %) and was not further analyzed.

**Table 2:** Association of HMGCR expression with tumor characteristics

	All patients median (IQR) or %	Missing	Patients with evaluable TMA	HMGCR expression-Intensity median (IQR) or %			
				No staining = 0	Weak = 1	Moderate/Strong = 2/3	*P*-value
n=	1026		885	113	555	217	
**pT**		0					0.77^a^
1	740 (72.1 %)		631 (71.3 %)	77 (68.1 %)	404 (72.8 %)	150 (69.1 %)	
2	269 (26.2 %)		238 (26.9 %)	34 (30.1 %)	140 (25.2 %)	64 (29.5 %)	
3	15 (1.5 %)		14 (1.6 %)	2 (1.8 %)	9 (1.6 %)	31 (1.4 %)	
4	2 (0.2 %)		2 (0.2 %)	0 (0 %)	2 (0.4 %)	0 (0 %)	
**Axillary node involvement**		2					0.020^b^
0	627 (61.2 %)		532 (60.2 %)	60 (53.1 %)	330 (59.6 %)	142 (65.7 %)	
1-3	306 (29.9 %)		270 (30.6 %)	37 (32.7 %)	177 (31.9 %)	56 (25.9 %)	
4+	91 (8.9 %)		81 (9.2 %)	16 (14.2 %)	47 (8.5 %)	18 (8.3 %)	
**Histological grade**		1					0.013^b^
I	252 (24.6 %)		203 (22.9 %)	20 (17.7 %)	126 (22.7 %)	57 (26.3 %)	
II	511 (49.9 %)		443 (50.1 %)	54 (47.8 %)	278 (50.1 %)	111 (51.2 %)	
III	262 (25.6 %)		239 (27.0 %)	39 (34.5 %)	151 (27.2 %)	49 (22.6 %)	
**Histological type**		1					0.512^a^
Mainly ductal	836 (81.6 %)		737 (83.4 %)	96 (85.0 %)	467 (84.3 %)	174 (80.2 %)	
Mainly lobular	121 (11.8 %)		97 (11.0 %)	11 (9.7 %)	55 (9.9 %)	31 (14.3 %)	
Other or mixed	66 (6.4 %)		50 (5.7 %)	6 (5.3 %)	32 (5.8 %)	12 (5.5 %)	
**Hormone receptor status**		2					
ER+	896 (87.5 %)		771 (87.2 %)	84 (74.3 %)	484 (87.4 %)	203 (93.5 %)	<0.0001^b^
PgR+	726 (70.9 %)		627 (70.9 %)	70 (61.9 %)	389 (70.2 %)	168 (77.4 %)	0.003^b^
HER 2 amplification*		59					0.009^b^
HER 2 positive	86 (11.7 %)		71 (11.1 %)	13 (21.0 %)	46 (11.2 %)	12 (7.2 %)	
HER 2 negative	601 (81.4 %)		527 (82.3 %)	47 (75.8 %)	340 (82.7 %)	140 (83.8 %)	

*Patients younger than 70 years of age and included as of November 2005 ^a^Chi-Square, ^b^Linear-by-linear

### Risk of early breast cancer related events

**Fig. 3:**
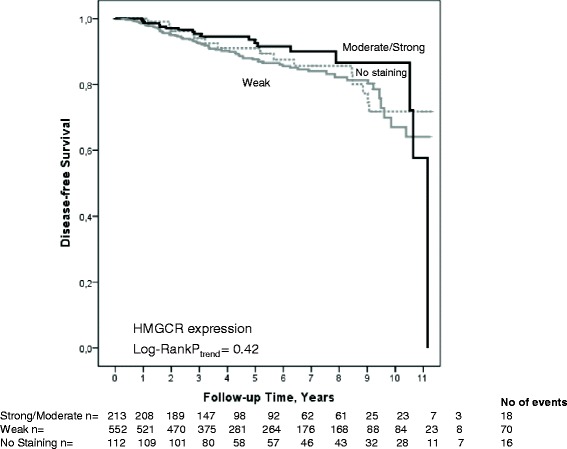
Kaplan-Meier estimate of DFS in relation to HMGCR expression. The number of patients at each follow-up is indicated. Since this study is an on-going study, the number of patients decreases with each follow-up

Patients were followed for up to 11 years. The median follow-up time was 5.0 years (interquartile range 3.0-7.2 years) for the 739 patients who were alive and still at risk at the last follow-up. The total number of patients with a breast cancer related event during follow-up was 104, of whom 68 patients were diagnosed with distant metastases. Of these 104 patients with a breast cancer related event, 53 patients subsequently died during follow-up. An additional 34 patients died without a prior recorded breast cancer related event. No significant association was observed between HMGCR expression and DFS either in univariable (Log-Rank *P*_trend_ = 0.42) (Fig. 3) or or multivariable models (Table 3). Likewise, no difference in DFS was observed between patients with any HMGCR expression and patients with HMGCR-negative tumors (Log-Rank *P*_trend_ = 0.90). In addition, no significant association was observed between HMGCR expression and distant metastasis-free survival (Log-Rank *P*_trend_ = 0.44), or overall survival (Log-Rank *P*_trend_ = 0.87). The results remained essentially the same in analyses restricted to patients with ER-positive tumors. Further stratification according to ER status, treatment (e.g., tamoxifen, aromatase inhibitors, radiotherapy, or chemotherapy), age or BMI failed to yield any significant associations between HMGCR expression and DFS in either univariable or multivariable models. The results remained essentially the same when excluding preoperative statin users. Similarly, the results did not differ when three patients with *in situ* breast cancer related events were excluded.

**Table 3:** Multivariable analysis of risk for breast cancer related events in relation to HMGCR status in all patients

		95 % CI	
	HR	Lower	Upper
HMGCR no staining	1.000		
HMGCR weak expression	1.389	0.792	2.436
HMGCR moderate/strong expression	1.103	0.548	2.218
Invasive tumor size ≥ 21 mm or muscular/skin involvement	2.041	1.329	3.133
Axillary nodal involvement	1.427	0.946	2.153
Histological grade III	1.292	0.789	2.115
ER status	0.596	0.316	1.125
PgR status	0.740	0.448	1.223
Age ≥ 50 years	0.647	0.416	1.005
BMI ≥ 25 kg/m^2^	1.305	0.867	1.966
Preoperative smoker	1.289	0.811	2.050

## Discussion

The main finding of this study was that moderate/strong HMGCR expression was significantly associated with several indolent tumor characteristics, including lower histological grade, ER and PgR positivity, HER2 negativity, and less axillary lymph node involvement. These findings are largely consistent with a previous study that reported an association between stronger HMGCR expression and small tumor size, low histological grade, low Ki67, and ER expression [1]. However, in the present study, no association was observed between tumor size and HMGCR expression, and Ki67 was not included in the analyses as this marker was not routinely analyzed until March 2009 [28].

Patients with tumors that expressed moderate/strong HMGCR were significantly older at the time of inclusion in the present study, and only one of the patients with strong HMGCR staining was less than 50 years of age. This finding is consistent with another study that studied premenopausal patients and reported no tumors with strong expression of this marker [3]. ER-positive tumors are more common in postmenopausal patients than in premenopausal patients [29]. In the present study, moderate/strong HMGCR expression was associated with ER positivity, indicating that there might be an association between age, HMGCR expression and ER-positive tumors. This association may be linked to 27-hydroxycholesterol (27HC), which is a primary cholesterol metabolite and a selective estrogen receptor modulator (SERM) exerting ER agonistic effects, as recently shown in a study of murine models [30, 31]. The study demonstrated how conversion of cholesterol to 27HC was necessary for effects on ER-positive breast cancer cells and how the actions of 27HC on tumor growth were dependent on ER. Those findings shed light on how 27HC may promote cancer growth and serve as the link between hypercholesterolemia and ER-positive breast cancer in postmenopausal women.

Normal cells can obtain cellular cholesterol in two ways; either via receptor-mediated uptake (low-density-lipoprotein receptor) or by synthesizing cholesterol through the mevalonate pathway and the activity of HMGCR. The normal cellular response to low intracellular cholesterol levels is to increase HMGCR activity to maintain an intact mevalonate pathway. However, tumor cells that fail to respond to this feed-back loop might have lost the checkpoint controls that maintain an intact pathway or may have a deregulated pathway [1, 32]. This dysregulation of the mevalonate pathway and HMGCR activity can contribute to the transformation involved in oncogenesis and may be essential for the metabolic transformation of tumor cells, at least in some cancers [32]. Therefore, potential biomarkers within the mevalonate pathway that could predict the response to statin treatment are of interest.

A previous study has reported that ER-negative breast cancer is less likely to arise among statin users and that ER-negative cell lines are more sensitive to statin inhibition than ER-positive cell lines [33]. In the present study, tumors that were negative for both ER and HMGCR had a higher histological grade significantly more often than tumors that were positive for these markers (data not shown). This association may reflect an inability of less differentiated cancer cells to maintain an intact mevalonate pathway. It was previously proposed that some cancer cells could be statin-sensitive and unable to maintain adequate levels of mevalonate end products when exposed to statins, resulting in apoptosis [32]. In contrast, statin-insensitive tumor cells demonstrate a feedback response similar to that of normal cells, in which HMGCR is up-regulated; this response may protect these cells from the anticancer effects of statins [32]. It is possible that well-differentiated cancer cells but not less differentiated cancer cells are capable of initiating this response. Further studies are needed to explain the role of HMGCR in breast cancer.

No significant association was observed between HMGCR expression and DFS in the present study. Two previous studies reported associations between recurrence-free survival and HMGCR expression [2, 3]. However, the median follow-up time of the present study was only 5.0 years, compared to median follow-up times of 10.7 years [2] and 13.9 years [3] in the previous studies. Moderate/strong HMGCR expression was strongly associated with ER-positive tumors. ER-positive tumors are known to relapse later than ER-negative tumors; because 87.5 % of the patients in the present study had ER-positive tumors, a longer follow-up time may be needed [34].

HMGCR expression was negative in 12.8 % of the cases in this study. The percentage of tumors with negative staining varied between 18 % and 52.7 % of cases in previous studies [1–3, 35]. However, these studies had fewer tumors that stained for HMGCR. In addition, one study included only premenopausal patients [3], which may have affected the results because HMGCR was significantly associated with age in the present study. Although the intensity varied between studies, the finding in the current study that HMGCR is expressed in the majority of the cells when present is consistent with other studies [2, 3].

HMGCR is differentially expressed and often overexpressed in tumor cells [18] and high expression appears to be associated with less aggressive tumor characteristics [1, 2]. Previous studies reported that HMGCR expression was a good prognostic marker [2, 3]. A previous window-of-opportunity study demonstrated that patients who were treated with statins for two weeks pre-operatively exhibited increased expression of HMGCR in the tumor and a reduced proliferation rate of Ki67 [35]. The increase of HMGCR expression that occurs after statin treatment indicate that statins affected the tumor either directly through inhibition of HMGCR and the mevalonate pathway within the tumor or indirectly through lowered circulating levels of cholesterol and in both cases, a negative feed-back loop resulting in elevated intratumoral HMGCR levels [32]. Associations of HMGCR expression with more favorable tumor characteristics and a prolonged survival have also been demonstrated in patients with other types of cancer such as colorectal cancer [36].

Some limitations of this study should be considered. One weakness of the present study may be that HMGCR expression was evaluated on TMAs rather than in whole slide tumor tissue sections. However, a previous study stained five whole slide tumor tissue sections for HMGCR and this marker was homogeneously expressed in all of the sections [1]. Therefore, we believe that the HMGCR results obtained from TMAs are representative. The BC-blood study is an ongoing, population-based prospective study, which limits the risk for recall bias. The most common reason that patients did not participate in the present study was the lack of available research nurses. A previous study demonstrated that the patients who did not participate had patient and tumor characteristics similar to those who did participate [28]. This similarity makes the findings generalizable for breast cancer patients at Skåne University Hospital in Lund, Sweden. The patients were never asked about ethnicity. However, the majority of the patients were ethnic Swedes. To the authors’ knowledge, the variation of HMGCR expression in cancer cells among different ethnic groups has not been investigated previously.

In conclusion, high HMGCR expression appears to be associated with less aggressive tumor characteristics in this population-based cohort of unselected primary breast cancer patients. Despite this finding, no association between HMGCR expression and short-term DFS was observed. Since previous studies had longer follow-up times, their findings can be neither confirmed nor rejected. Further studies and a prolonged follow-up time are needed to evaluate HMGCR as a prognostic and treatment-predictive marker.

## Abbreviations

ATC: Anatomic Therapeutic Chemical
BMI: Body mass index
DFS: Disease-free survival
ER: Estrogen receptor
HER2: Human epidermal growth factor 2
HMGCR: 3-hydroxy-3-methylglutaryl-coenzyme A reductase
27-OHC: 27-hydroxycholesterol
PgR: Progesterone receptor
REMARK: Recommendations for tumour MARKer prognostic studies
TMA: Tissue micro array
